# Interplay between Position-Dependent Codon Usage Bias and Hydrogen Bonding at the 5ʹ End of ORFeomes

**DOI:** 10.1128/mSystems.00613-20

**Published:** 2020-08-11

**Authors:** Juan C. Villada, Maria F. Duran, Patrick K. H. Lee

**Affiliations:** a School of Energy and Environment, City University of Hong Kong, Kowloon, Hong Kong SAR, China; University of Pennsylvania

**Keywords:** codon variants, transcription efficiency, DNA unwinding, resource allocation, energy efficiency

## Abstract

Codon usage bias exerts control over a wide variety of molecular processes. The positioning of synonymous codons within coding sequences (CDSs) dictates protein expression by mechanisms such as local translation efficiency, mRNA Gibbs free energy, and protein cotranslational folding. In this work, we explore how codon usage affects the position-dependent content of hydrogen bonding, which in turn influences energy requirements for unwinding double-stranded DNA (dsDNA). We categorized codons according to their hydrogen bond content and found differential effects on hydrogen bonding encoded by codon variants. The specific positional disposition of codon variants within CDSs creates a ramp of hydrogen bonding at the 5ʹ end of the ORFeome in Escherichia coli. CDSs occupying the first position of operons are subjected to selective pressure that reduces their hydrogen bonding compared to internal CDSs, and highly transcribed CDSs demand a lower maximum capacity of hydrogen bonds per codon, suggesting that the energetic requirement for unwinding the dsDNA in highly transcribed CDSs has evolved to be minimized in E. coli. Subsequent analysis of over 14,000 ORFeomes showed a pervasive ramp of hydrogen bonding at the 5ʹ end in *Bacteria* and *Archaea* that positively correlates with the probability of mRNA secondary structure formation. Both the ramp and the correlation were not found in *Fungi*. The position-dependent hydrogen bonding might be part of the mechanism that contributes to the coordination between transcription and translation in *Bacteria* and *Archaea*. A Web-based application to analyze the position-dependent hydrogen bonding of ORFeomes has been developed and is publicly available (https://juanvillada.shinyapps.io/hbonds/).

**IMPORTANCE** Redundancy of the genetic code creates a vast space of alternatives to encode a protein. Synonymous codons exert control over a variety of molecular and physiological processes of cells mainly through influencing protein biosynthesis. Recent findings have shown that synonymous codon choice affects transcription by controlling mRNA abundance, mRNA stability, transcription termination, and transcript biosynthesis cost. In this work, by analyzing thousands of *Bacteria*, *Archaea*, and *Fungi* genomes, we extend recent findings by showing that synonymous codon choice, corresponding to the number of hydrogen bonds in a codon, can also have an effect on the energetic requirements for unwinding double-stranded DNA in a position-dependent fashion. This report offers new perspectives on the mechanism behind the transcription-translation coordination and complements previous hypotheses on the resource allocation strategies used by *Bacteria* and *Archaea* to manage energy efficiency in gene expression.

## Abstract (precis)

Redundancy of the genetic code creates a vast space of alternatives to encode a protein. Synonymous codons exert control over a variety of molecular and physiological processes of cells mainly through influencing protein biosynthesis. Recent findings have shown that synonymous codon choice affects transcription by controlling mRNA abundance, mRNA stability, transcription termination, and transcript biosynthesis cost. In this work, by analyzing thousands of *Bacteria*, *Archaea*, and *Fungi* genomes, we extend recent findings by showing that synonymous codon choice, corresponding to the number of hydrogen bonds in a codon, can also have an effect on the energetic requirements for unwinding double-stranded DNA in a position-dependent fashion. This report offers new perspectives on the mechanism behind the transcription-translation coordination and complements previous hypotheses on the resource allocation strategies used by *Bacteria* and *Archaea* to manage energy efficiency in gene expression.

## INTRODUCTION

Codon usage controls protein synthesis through a variety of mechanisms (1, 2). A number of classic works have established the links between codon usage and mRNA translation (3–5), with important insights into the physiological consequences of synonymous mutations (6, 7). The specific arrangement of synonymous codons in coding sequences (CDSs) has been shown to serve as a regulatory mechanism for translation dynamics (8) and protein cotranslational folding (9). In particular, the 5ʹ-end region of CDSs has strong effects on translation where synonymous codon choice is associated with targeting efficiency of signal peptides (10), ramping of translation efficiency (11), local folding energy (12), modulated protein expression (13), and recognition of nascent peptides by the signal recognition particle (14).

Similarly to translation, codon usage bias has been associated with transcriptional selection (15) and optimization of transcription efficiency (16). Recent reports support the idea that codon variants also define the energy and cellular resources required for transcript biosynthesis (17–20) and the speed of transcript elongation (21). However, in contrast to translation, the potential links between position-dependent codon usage bias at the 5ʹ end of CDSs and transcription have yet to be thoroughly investigated as it is difficult to disentangle the effects operating at the level of transcription from those operating at the level of translation, where position-dependent codon usage bias is known to have an effect (3–5).

During transcription, helicases melt the hydrogen bonds in double-stranded DNA (dsDNA) (22–25) to expose the single-stranded DNA (ssDNA) template sequence, while RNA polymerase produces the RNA molecule (26). Although the role of helicase can be active or passive (27), the dsDNA unwinding process requires energy (28) and successful unwinding of the dsDNA is a determinant in preventing abortive transcription and translation initiation (29). In this work, we explore whether the previously established position-dependent arrangement of codons can also create a position-dependent energetic requirement to unwind dsDNA by controlling the number of hydrogen bonds. Our central hypothesis stems from the fact that increased GC content of a gene increases the number of hydrogen bonds in its dsDNA, thereby demanding higher unwinding energy (30).

Here, by first analyzing the ORFeome (the set of all CDSs in a genome) of Escherichia coli as a model and subsequently extending the investigation to a more comprehensive set of over 14,000 ORFeomes, we provide genomic evidence that codon usage bias creates an exponentially increasing ramp of hydrogen bonding at the 5ʹ end of CDSs in *Bacteria* and *Archaea*. The findings in this study are not intended to provide evidence for stronger positional selection of codons for transcription efficiency over the well-established theories of position-dependent codon selection in translation efficiency (11) and mRNA secondary structure (12). Instead, our results suggest that as another layer of a potential biological role, position-dependent codon usage bias creates a position-dependent energetic requirement for unwinding dsDNA. This report provides novel insights into the evolution of molecular traits and the trade-offs between the genetic code and the physiology of organisms.

## RESULTS

### Effects of codon variants on hydrogen bonding and its positional dependency at the 5ʹ end of the E. coli ORFeome.

**FIG 1:**
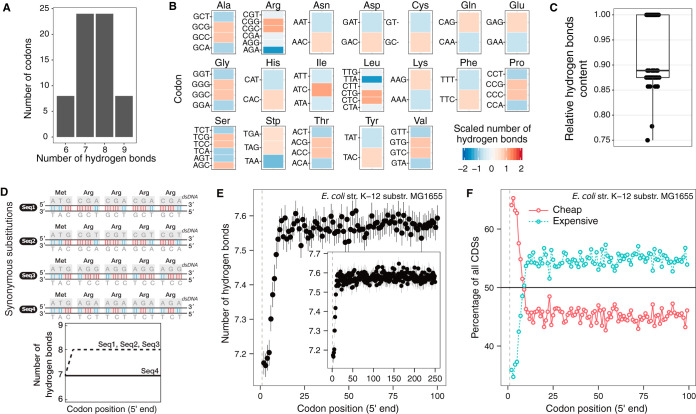
The trade-off between codon usage bias and the number of hydrogen bonds. (A) Frequency of codons according to the number of hydrogen bonds a codon can contain. (B) Number of hydrogen bonds (value scale is shown) for each amino acid by codon. Synonymous codon choices can reduce or increase the number of hydrogen bonds of each amino acid. Scaled values were calculated by centering and scaling the number of hydrogen bonds of codons that code for the same amino acid. (C) Hydrogen bonding content of codons relative to the maximum possible content among synonymous codons. Relative content values were calculated as the number of hydrogen bonds of each codon divided the maximum number of hydrogen bonds per amino acid. (D) Toy example illustrating how synonymous mutations in CDSs can create different distributions of position-dependent hydrogen bonds. (E) The number of hydrogen bonds gradually increases in the ORFeome of E. coli. The data shown correspond to the mean and 95% confidence interval of the mean with 1,000 bootstraps. The dashed line indicates the position of the start codon. The inset shows the number of hydrogen bonds up to the 250th codon position. (F) Usage of cheap and expensive codons based on the number of hydrogen bonds along CDSs of E. coli.

We began our analysis by categorizing codons according to their hydrogen bond content (Fig. 1). The number of hydrogen bonds in a codon is directly coupled to the GC content of a codon due to the Watson-Crick base pairing of nucleotides (31). Each codon can contain six to nine hydrogen bonds, but most codons tend to have seven or eight (Fig. 1A). All degenerate amino acids have choices for codons with different numbers of hydrogen bonds (Fig. 1B), and the relative content of hydrogen bonding of a codon can be decreased by 25% according to the synonymous codon choice (Fig. 1C). The range of choices for hydrogen bonding becomes wider in accordance with position-dependent codon usage bias, where the overall and local hydrogen bond composition of a CDS can be fine-tuned by introducing synonymous mutations (Fig. 1D).

All CDSs in the ORFeome of E. coli K-12 substrain MG1655 were analyzed to test whether the number of hydrogen bonds follows a positional dependency at the 5ʹ end. The mean number of hydrogen bonds in each codon position was calculated. We observed that the number of hydrogen bonds per codon gradually increased in a position-dependent manner until about the 15th codon position. After this codon position, the number of hydrogen bonds converged to levels of carrying capacity that remained similar until the 250th codon position (Fig. 1E). Subsequently, we discretized codons into the following two groups according to their hydrogen bond content: “cheap” codons (with six or seven hydrogen bonds) and “expensive” codons (with eight or nine bonds). We observed that the members of the group of cheap codons are utilized with high (∼65%) frequency and that their use then decreases gradually in a position-dependent manner until an equilibrium is reached at about the 15th codon position (Fig. 1F). From the 15th codon position to the 100th, the frequencies of utilization of cheap and expensive codons do not vary by more than ∼5%, with cheap codons appearing much less frequently than expensive codons (Fig. 1F).

Taken together, these results show that the choice of different synonymous codons can affect hydrogen bonding and that the E. coli ORFeome apparently uses this flexibility in a way that smoothly increases the energetic requirement for unwinding the dsDNA molecule in CDSs.

### Lower hydrogen bonding at the first CDS of operons in E. coli.

One biological interpretation of the observed position-dependent hydrogen bonding is that it may favor CDS transcription according to the modulated efficiency of dsDNA unwinding. Thus, evolution might reflect differential selective forces for hydrogen bonding optimization acting on the CDSs of operons with more than one CDS. Specifically, if hydrogen bonding has an effect on transcription, the first CDS within an operon, being closest to the beginning of the transcriptional unit, should be better optimized for lower hydrogen bonding than internal CDSs.

**FIG 2:**
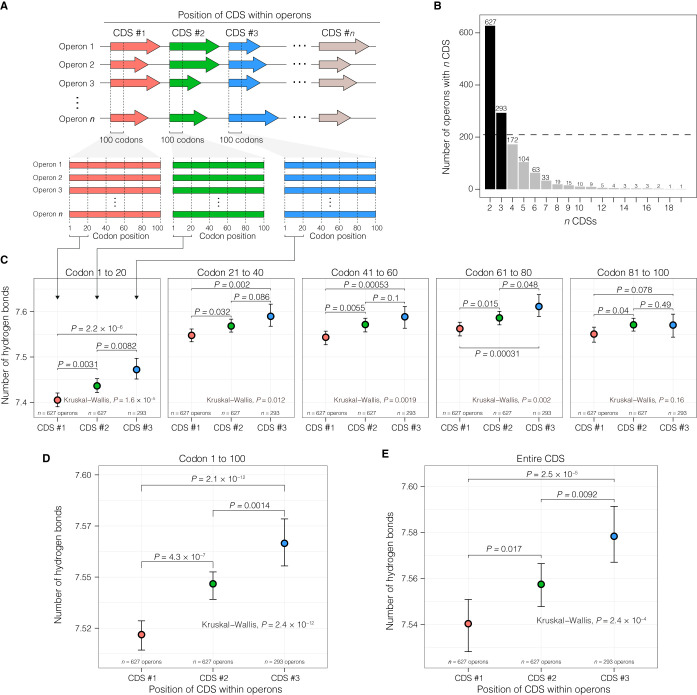
Position-dependent hydrogen bonding in operons of E. coli. (A) Schematic of the framework applied to study selection for hydrogen bonding in CDSs within an operon. (B) Histogram of the number of CDSs found in operons of E. coli after removing CDSs that are not part of operons. The dashed line demarcates one third of the number of operons with two CDSs. (C to E) Comparison of the numbers of hydrogen bonds according to CDS position within operons delineated by different CDS lengths for various codons (C), codons 1 to 100 (D), and the entire CDS. Means and the 95% confidence interval of the means are shown. The *P* value at the top of the brackets indicates the statistical significance of the Wilcoxon test results.

To test this hypothesis, the number of hydrogen bonds of CDSs according to the position they occupy within an operon in E. coli was quantified (Fig. 2A). Only operons containing two or more CDSs were analyzed, and the downstream analyses focused on the first three CDS positions within an operon as the number of operons with more than three CDSs is low (less than a third of the number of operons with two CDSs) (Fig. 2B). We observed that CDSs in the first position within an operon (i.e., CDS 1) had a significantly lower number of hydrogen bonds (Wilcoxon test, *P < *0.05) than the internal CDSs (i.e., CDS 2 and CDS 3) in the majority of the codon positions along the length of a CDS (Fig. 2C).

The preference for a lower number of hydrogen bonds appeared weaker downstream of the 20th codon position as the difference in the hydrogen bonding between CDS 1 and subsequent CDSs became consistently and gradually less significant as indicated by both the pairwise comparisons (Wilcoxon test) and group rank differences according to CDS position (Kruskal-Wallis test) (Fig. 2C). In the codons in positions 81 to 100, the number of hydrogen bonds between CDS positions was not significant (Kruskal-Wallis test, *P > *0.05). The number of hydrogen bonds in CDS 2 was significantly lower (Wilcoxon test, *P = *0.0082) than that in CDS 3 primarily in codon positions 1 to 20 (Fig. 2C). However, differences in hydrogen bonding based on CDS position were found to be preserved in comparisons of codon positions from 1 to 100 (Fig. 2D) and over the entire length of a CDS (Fig. 2E). Together, these results suggest that the proposed transcriptional efficiency hypothesis favors the beginning of the transcription unit in E. coli.

### Highly transcribed CDSs require a lower maximum capacity of hydrogen bonds per codon in E. coli.

**FIG 3:**
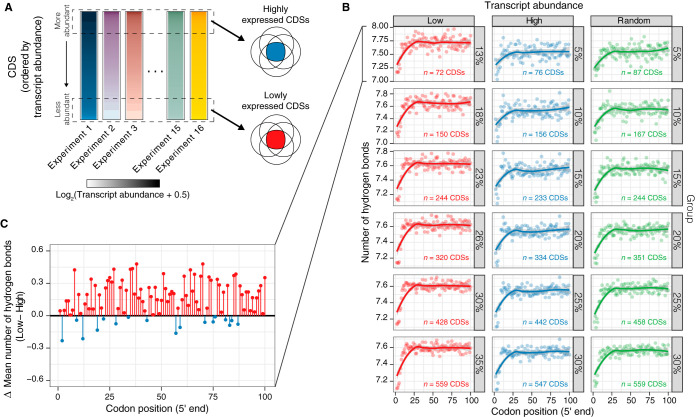
Relationships between position-dependent hydrogen bonding and transcript abundances in E. coli. (A) Schematic of the framework applied to study the number of hydrogen bonds in different groups of CDSs according to transcript abundances in 16 different RNA-Seq experiments. (B) Position-dependent hydrogen bonding as a function of codon position for six different groups corresponding to high and low levels of expression. Similar numbers of CDSs were randomly selected at high and low levels of expression regardless of transcript abundances for comparison in each group. The gray-shaded region represents the 95% confidence interval of the predicted mean of the locally estimated scatterplot smoothing (LOESS) function. (C) Differences in the mean numbers of hydrogen bonds by codon position between the bottom 13% and top 5% of transcript abundances.

An alternative approach to assess the proposed association between position-dependent hydrogen bonding and dsDNA unwinding energy is to study whether there are differences in hydrogen bonding between CDSs with different expression levels. We hypothesized that if CDSs prefer codons with a lower number of hydrogen bonds at the 5′ end to optimize transcription, the position-dependent hydrogen bonding might be differentiable according to transcript abundances. By analyzing the transcriptome sequencing (RNA-Seq) data of E. coli generated under 16 different sets of conditions (32) as illustrated in Fig. 3A, we found that highly transcribed CDSs required lower levels of hydrogen bonding (Fig. 3B) and that the level of hydrogen bonding was generally lower in most codon positions from 1 to 100 (Fig. 3C) than with the minimally transcribed CDSs. The differences in the levels of hydrogen bonding increased with the level of disparity in transcript abundances between highly and minimally expressed CDSs (Fig. 3B), suggesting that a preference for lower numbers of hydrogen bonds helps to optimize transcription (Fig. 3B). The position-dependent hydrogen bonding of randomly selected CDSs indicated that most CDSs still exhibited a ramp regardless of transcript abundance (Fig. 3B). Overall, we observed that highly transcribed CDSs in E. coli required a lower maximum capacity of hydrogen bonds per codon, suggesting that the energetic requirement to unwind the dsDNA is lower for highly transcribed CDSs than for minimally transcribed CDSs (Fig. 3).

### Distinguishing position-dependent hydrogen bonding from translation-related and mRNA secondary structure-based phenomena in E. coli and Saccharomyces cerevisiae.

In order to support the hypothesis of the transcriptional relevance of position-dependent hydrogen bonding and to distinguish it from the already known translation-related and mRNA secondary structure-based hypotheses, we assessed the potential relationships between the position-dependent hydrogen bonding and the metrics traditionally used in codon usage bias studies for E. coli and S. cerevisiae (to gain insights into potential differences between *Bacteria* and *Archaea* and eukaryotes). The metrics computed as a function of the codon position were (i) the frequency of preferred codons (determined using relative synonymous codon usage [RSCU] data), (ii) mRNA secondary structure folding (using the probability of base pairing), (iii) codon optimality (using the codon adaptation index [CAI]), (iv) translation efficiency (using the tRNA adaptation index [tAI]), and (v) hydrogen bonding.

**FIG 4:**
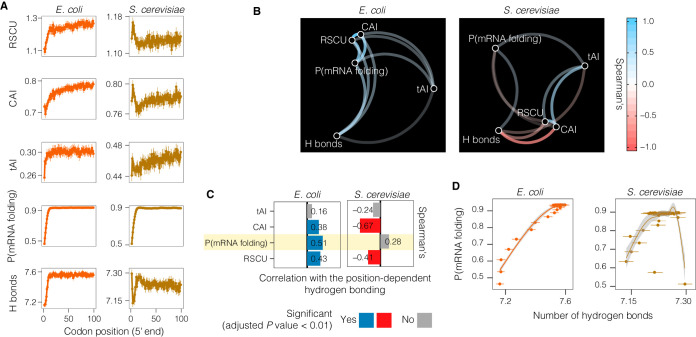
Position-dependent codon usage bias and its relationship with hydrogen bonding in the ORFeomes of E. coli and S. cerevisiae. (A) Analysis of five different metrics used to study codon usage bias as a function of codon position. Means and 95% confidence intervals of the means are shown. (B) Correlation network analysis among all the metrics analyzed. Spearman’s correlation coefficient is reported in this analysis. (C) Spearman correlation tests illustrating the relationships between hydrogen bonding with the five metrics examined. The statistical significance of the correlation was adjusted for multiple testing. (D) Generalized additive model (GAM) describing position-dependent hydrogen bonding as a function of the probability of mRNA secondary structure formation. The gray-shaded region represents the 95% confidence interval of the predicted mean.

We observed a ramp in all the codon usage metrics, mRNA folding, and hydrogen bonding as a function of codon position in E. coli (Fig. 4A). In contrast, the results obtained for S. cerevisiae showed that hydrogen bonding and mRNA secondary structure formation appeared unrelated (Fig. 4A). In order to understand the potential associations among all the computed metrics, a correlation network analysis was conducted (Fig. 4B). We found that hydrogen bonding significantly (adjusted *P < *0.01) and strongly (Spearman’s ρ = 0.51) correlated with the mRNA secondary structure in E. coli but not in S. cerevisiae (Spearman’s ρ = 0.28) (Fig. 4C). Consistently, the ramps found in mRNA secondary structure and hydrogen bonding were found to be strongly related in the first 15 to 20 codons only in E. coli (Fig. 4D). Overall, the observed correlation suggests that selection acts to maintain tightly associated ramps in mRNA secondary structure and hydrogen boding only in E. coli (Fig. 4).

In order to assess whether these observations could be extended to other microorganisms, we deployed the same analyses on a set of model *Bacteria*, *Archaea*, and *Fungi* (see Fig. S1 in the supplemental material). Although the conclusions remained largely the same for the other ORFeomes, there were some differences. For example, similarly to the results seen with E. coli, a ramp was also observed in all the codon usage metrics and hydrogen bonding as a function of codon position in the archaeon Haloferax volcanii, but this was not the case for the other model ORFeomes analyzed (Fig. S1A). Although the bacterium “*Candidatus* Methylacidiphilum kamchatkense” and the archaeon Methanosarcina acetivorans did not show a clear positional dependency on the frequency of preferred codons (RSCU), codon optimality (CAI), and translation efficiency (tAI), mRNA folding and hydrogen bonding showed a ramp (Fig. S1A), indicating that the hydrogen bonding phenomenon is distinguishable from the other codon usage-related phenomena in these organisms. In general, position-dependent hydrogen bonding was found to be tightly related to the mRNA secondary structure formation in the model *Bacteria* and *Archaea* but not in the model eukaryotes (Fig. S1B to D).

10.1128/mSystems.00613-20.1 FIG S1 Extended analysis of position-dependent codon usage bias and its relationship with hydrogen bonding in the ORFeomes of eight model organisms. (A) Analysis of five different metrics used to study codon usage bias as a function of codon position. Means and 95% confidence intervals of the means are shown. (B) Correlation network analysis among all the metrics analyzed (Spearman’s correlation coefficient is reported). (C) Correlation tests (Spearman and Pearson) illustrating the relationships between hydrogen bonding and the five metrics examined. The statistical significance of the correlation was adjusted for multiple testing. (D) Generalized additive model (GAM) describing position-dependent hydrogen bonding as a function of the probability of mRNA secondary structure formation. The gray-shaded region represents the 95% confidence interval of the predicted mean. Download FIG S1, PDF file, 2 MB. Copyright © 2020 Villada et al. 2020 Villada et al. This content is distributed under the terms of the Creative Commons Attribution 4.0 International license.

### Modeling the hydrogen bonding ramp in E. coli.

**FIG 5:**
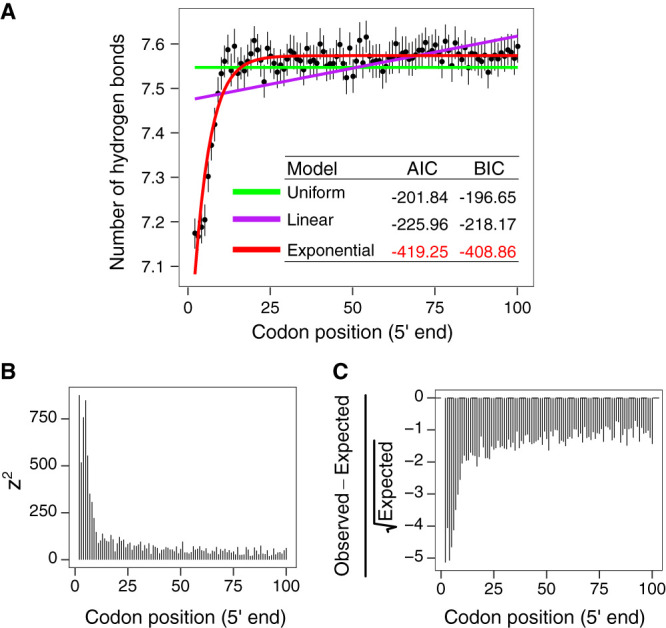
Modeling the ramp of the number of hydrogen bonds and test of selection against uniform distribution of the number of hydrogen bonds per codon in E. coli. (A) Three mathematical models were fitted to the hydrogen bonding data. The bounded exponential model with three parameters (red line) produced the best fit to the observed data. (B) *z*^2^ value per codon position according to the χ^2^ statistic. The higher the *z*^2^ value, the higher the level of selection acting against uniform distribution. (C) χ-gram value (equation 3) per codon position.

After investigating the biological relevance of the ramp of hydrogen bonding as a function of transcriptional unit (Fig. 2) and gene expression (Fig. 3) as well as identifying its association with the mRNA secondary structure formation as a potential genomic signal of the coupling between transcription and translation in *Bacteria* and *Archaea* (Fig. 4), we then sought to model and characterize the ramp in E. coli. We tested three mathematical functions to model the mean number of hydrogen bonds per codon as a function of codon position. According to Akaike information criterion (AIC) and Bayesian information criterion (BIC) data, the bounded exponential model with three parameters (initial content, rate, and carrying capacity) produced the best fit (Fig. 5A). The fitness of the model showed that the number of hydrogen bonds per codon follows an exponential function of codon position with a positive rate that has a ramp-like shape at the 5ʹ end of CDSs.

### Testing the selection for reduced hydrogen bonding at the 5ʹ end in E. coli.

After determining that the ramp of hydrogen bonding can be better fitted by an exponential model, we further tested whether selection acts, through position-dependent codon usage bias, against uniform distribution of hydrogen bonds per codon along CDSs in E. coli. To test this hypothesis, we applied codon shuffling techniques (33, 34) to generate 200 simulated ORFeomes of E. coli that contained random synonymous mutations. The codon-shuffled ORFeomes were used as a null model to test selection against uniformity using the χ^2^ statistic (33, 34).

The *z*^2^ value (from the χ^2^ statistic) per codon position showed that selection acted against uniform distribution of the number of hydrogen bonds and that selection was noticeably stronger at the 5ʹ end of the E. coli ORFeome (Fig. 5B). Finally, we investigated the direction of selection acting on the 5ʹ end of the E. coli ORFeome. To assess the selection direction, we computed the value for the χ-gram and found that selection acted to reduce the number of hydrogen bonds at the 5ʹ end of CDSs in the E. coli ORFeome in a position-dependent manner (Fig. 5C).

### Position-dependent hydrogen bonding consistently correlates with mRNA structure folding in *Bacteria* and *Archaea* but not *Fungi*.

As local reduction of base pairing probability in mRNA facilitates translation initiation (35), this matched the observed region of reduced hydrogen bonding in the selected model *Bacteria and Archaea* (Fig. 4; see also Fig. S1). Next, we tested whether the observed correlations between hydrogen bonding in CDSs and formation of the mRNA secondary structure could be a genomic signal in diverse genera of *Bacteria* and *Archaea*, but not eukaryotes, as part of the molecular mechanism that optimizes the coordination between transcription and translation (36). The expectation is that for genes of organisms whose transcription and translation are coupled in space and time (i.e., *Bacteria* and *Archaea*), the significant and strong positive correlation between the position-dependent mRNA secondary structure formation and hydrogen bonding should be found to be universally conserved. In contrast, the correlation in eukaryotes should be insignificant or weaker.

**FIG 6:**
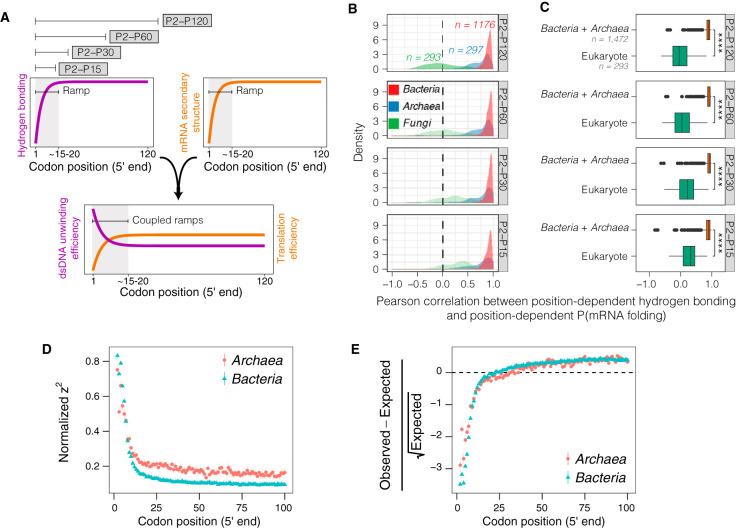
Selection for position-dependent hydrogen bonding and its association with the probability of mRNA secondary structure formation in *Bacteria* and *Archaea* of the representative data set. (A) Schematic illustrating the region of codon positions analyzed and the characteristics of the hydrogen bonding and mRNA secondary structure ramps. (B) Distributions of the Pearson correlations between position-dependent probabilities of mRNA folding and hydrogen bonding. (C) Hypothesis testing (two-tailed *t* test) of the differences between *Bacteria* and *Archaea* and eukaryotes in the distributions of correlations between position-dependent probabilities of mRNA folding and hydrogen bonding. ****, *P < *1 × 10^−16^. (D) Test of selection against uniform distribution of the number of hydrogen bonds per codon in all ORFeomes of the representative data set. Means and 95% confidence intervals of the means with 1,000 bootstraps of the *z*^2^ value normalized by *min-max* normalization (equation 4). (E) Means and 95% confidence intervals of the means with 1,000 bootstraps of the scaled χ-gram value (equation 3).

To investigate this issue, the position-dependent probabilities of pairing of mRNA and position-dependent hydrogen bonding of ∼1,700 ORFeomes in the representative data set were computed. We discretized the correlation analysis by different regions of codon position (Fig. 6A) and found that the positive and strong correlation was conserved in *Bacteria* and *Archaea* regardless of the codon position region (Fig. 6B). However, despite an increase in the Pearson’s (Fig. 6B) and Spearman’s (Fig. S2A) median correlation values in *Fungi* as the codon position region was shortened, the correlation values were found to be <0.5 in the best-case scenario and much lower than those seen in *Bacteria* and *Archaea*. Overall, the correlation between the position-dependent probability of pairing of mRNA and position-dependent hydrogen bonding in *Bacteria* and *Archaea* is significantly stronger than that seen with eukaryotes (Fig. 6C; see also Fig. S2B). While these two metrics are expected to correlate positively with one another, the consistently strong associations observed for *Bacteria* and *Archaea* provide new insights into the evolutionary coupling of transcription and translation through the position-dependent optimization of hydrogen bonding and mRNA pairing probability. Accordingly, we further investigated whether evolution preserves position-dependent hydrogen bonding in *Bacteria* and *Archaea*. The results of the test for selection against uniform distribution of hydrogen bonds per codon along CDSs on every ORFeome in the representative data set indicated that both the strength of the selection (Fig. 6D; see also Fig. S3A) and the direction of the selection (Fig. 6E; see also Fig. S3B) are conserved in *Bacteria* and *Archaea*.

10.1128/mSystems.00613-20.2 FIG S2 Extended analysis of the potential associations between position-dependent probability of mRNA folding and hydrogen bonding for ORFeomes in the representative data set. (A) Distributions of the correlations (Pearson and Spearman) between position-dependent probability of mRNA folding and hydrogen bonding. (B) Hypothesis testing (two-tailed *t*-test) of the differences between *Bacteria* and *Archaea* and eukaryotes in the distribution of correlations between position-dependent probability of mRNA folding and hydrogen bonding. ****, *P < *1 × 10^−16^. (C) Analysis of Pearson correlation distribution by taxonomic class. The codon positions tested correspond to the P2-to-P120 region. The panel is vertically ordered by the median value of the correlation distribution per taxonomic class. Genomic features of GC and GC_3_ content and mutational bias were mapped to each taxonomic class. Download FIG S2, PDF file, 1.6 MB. Copyright © 2020 Villada et al. 2020 Villada et al. This content is distributed under the terms of the Creative Commons Attribution 4.0 International license.

10.1128/mSystems.00613-20.3 FIG S3 Detailed illustration of the ramp of selection against uniform distribution of the number of hydrogen bonds per codon for every ORFeome in the representative data set. (A) Normalized *z*^2^ and (B) scaled χ-gram of each ORFeome (∼1,500 in total). Columns correspond to the codon position (from 2nd to 100th), and rows correspond to individual organisms. The 5′ end (first ∼15 codon positions) of CDSs shows the highest level of selection. Download FIG S3, PDF file, 1.8 MB. Copyright © 2020 Villada et al. 2020 Villada et al. This content is distributed under the terms of the Creative Commons Attribution 4.0 International license.

Finally, we studied if the distribution of correlations between the position-dependent pairing probability of mRNA and position-dependent hydrogen bonding is associated with specific taxonomic classes and whether these classes show similar patterns of genomic GC and GC_3_ content and mutational bias (i.e., GC_3_/GC) (Fig. S2C). As an outlier with respect to the correlation, the members of the bacterial class *Mollicutes* were found to contain a set of ORFeomes for which the correlation was weakly positive (Fig. S2C). *Mollicutes* also showed the lowest genomic GC and GC_3_ content in the set of bacterial and archaeal ORFeomes analyzed (Fig. S2C). All other *Bacteria* and *Archaea* classes showed equally strong correlations but variable genomic GC and GC_3_ content and mutational biases (Fig. S2C). Interestingly, the three fungal groups showing the highest median of the correlation distribution corresponded to the three classes with the lowest genomic GC and GC_3_ content and a mutational bias value of <1.0 (Fig. S2C). The *Fungi* classes *Malasseziomycetes* and *Tremellomycetes* showed the strongest correlations between the position-dependent pairing probability of mRNA and position-dependent hydrogen bonding, but these correlations were negative, and no associations were found with the GC and GC_3_ content and mutational bias (Fig. S2C). Overall, the results from this representative data set showed that position-dependent hydrogen bonding consistently correlates with mRNA structure folding in *Bacteria* and *Archaea* but not eukaryotes and that selection against uniform distribution of codons within CDSs acts on these bacterial and archaeal ORFeomes to reduce the number of hydrogen bonds in the first codons of CDSs.

### Modeling the hydrogen bonding ramp in ORFeomes of *Bacteria*, *Archaea*, and *Fungi*.

**FIG 7:**
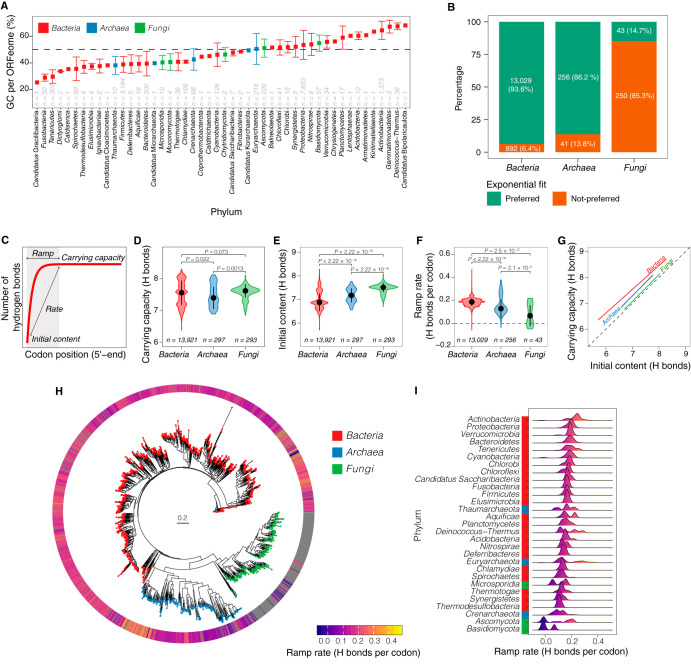
Characteristics of the hydrogen bonding ramp in ORFeomes of the comprehensive data set (A to G) and phylogenomics of the ramp rate in the representative data set (H to I). (A) Mean GC content of all CDSs in each ORFeome by phyla. (B) Percentages of ORFeomes that fitted the bounded exponential model. (C) Graphical representation of the three estimated parameters for the ramp detected by the bounded exponential model. (D to F) Comparison of the distributions of estimated (D) carrying capacities of hydrogen bonds, (E) initial content of the number of hydrogen bonds, and (F) rates of the ramp between *Bacteria*, *Archaea* and *Fungi*. In all panels, reported *P* values correspond to the Wilcox test adjusted for multiple testing, and *n* is the number of ORFeomes for which the parameter could be successfully estimated. (G) Linear regression model with the initial number of hydrogen bonds as independent variable and the carrying capacity of hydrogen bonds as dependent variable. (H) Whole-genome phylogenetic tree of the three domains of life with the ramp rate estimated for each ORFeome mapped to the tree. (I) Distribution of the ramp rate by phyla in the phylogenetic tree. Phyla are sorted by the median of the ramp rate of all ORFeomes in each phylum. For visual clarity, phyla with fewer than three ORFeomes are not shown.

After we had successfully modeled the hydrogen bonding ramp in E. coli (Fig. 5A) and identified its association with the mRNA secondary structure formation as a potential genomic signal of the coupling between transcription and translation in *Bacteria* and *Archaea* but not in eukaryotes (Fig. 6A to C), we further investigated whether the bounded exponential ramp model can be universally fitted to diverse ORFeomes. To explore this issue, we compiled a comprehensive data set with ∼14,500 ORFeomes that included *Bacteria*, *Archaea*, and *Fungi* from diverse phyla (Fig. 7A). The data set comprised ORFeomes with various numbers of CDSs (Fig. S4A), total lengths (Fig. S4B), mean CDS lengths (Fig. S4C), diverse GC_3_/GC ratios (Fig. S4D), and different mutational biases per phylum (Fig. S4E). We analyzed the position-dependent number of hydrogen bonds per codon of each ORFeome and found that in most *Bacteria* and *Archaea* (94% of *Bacteria* and 86% of *Archaea*), the number of hydrogen bonds per codon position could be successfully fitted by the bounded exponential model whereas the fit of this model was unsuccessful in most *Fungi* (85%) (Fig. 7B). Instead, the linear model produced a better fit for most of the fungal ORFeomes (Fig. S5A) and the subset of ORFeomes successfully fitted by the bounded exponential model was not monophyletic (Fig. S5B). We further investigated differences between the groups that successfully and unsuccessfully fitted the bounded exponential model, and only two significant different features were observed (Fig. S6). First, the total ORFeome lengths tended to differ between the two modeled groups in *Bacteria* and *Fungi* (Fig. S6A, *P < *0.001); second, the mean lengths of CDS per genome were significantly different in *Bacteria* (Fig. S6B, *P < *0.001). No differences were found for GC_3_/GC ratios (Fig. S6C). Scrutinized by phylum, only *Aquificae* and *Nitrospirae* showed major differences in genomic GC content (Fig. S6D) and mutational bias (Fig. S6E) between the two modeled groups (caused by outlier ORFeomes). For the outliers ORFeomes that could not be successfully modeled by the bounded exponential model, it was found that they had a relatively higher GC content and a higher GC_3_/GC ratio.

10.1128/mSystems.00613-20.4 FIG S4 Characteristics of the ORFeomes in the comprehensive data set. (A) Distributions of the number of CDSs per ORFeome. (B) Total ORFeome length (in base pairs [bp]). (C) Mean length of CDSs in each genome. (D) Distribution of mutational biases in each ORFeome computed as the ratio of GC_3_ to GC content in *Bacteria*, *Archaea*, and *Fungi*. (E) Mean ratio of GC_3_ content to GC content for all CDSs in each ORFeome grouped by phyla. Error bars represent 1 standard deviation. Download FIG S4, PDF file, 0.3 MB. Copyright © 2020 Villada et al. 2020 Villada et al. This content is distributed under the terms of the Creative Commons Attribution 4.0 International license.

10.1128/mSystems.00613-20.5 FIG S5 Model fitting of fungal ORFeomes in the comprehensive data set. (A) Frequency of the best fitted model in fungal ORFeomes (*n *= 293) according to the Akaike information criterion (AIC) and Bayesian information criterion (BIC). (B) Families of fungal ORFeomes successfully and unsuccessfully fitted by the use of the bounded exponential model. Download FIG S5, PDF file, 0.2 MB. Copyright © 2020 Villada et al. 2020 Villada et al. This content is distributed under the terms of the Creative Commons Attribution 4.0 International license.

10.1128/mSystems.00613-20.6 FIG S6 Characteristics of ORFeomes in the comprehensive data set delineated by the fit of the bounded exponential model. (A) Total ORFeome length (in base pairs [bp]). (B) Mean length (in bp) of CDSs in each ORFeome. (C) Distribution of mutational biases found in each ORFeome computed as the ratio of GC_3_ content to GC content in *Bacteria*, *Archaea*, and *Fungi*. All the indicated *P* values correspond to hypothesis testing of the differences between the successful and unsuccessful groups with the two-tailed unpaired *t*-test. (D and E) Mean GC content (D) and mean ratio of GC_3_ content to GC content (E) for all CDSs in each ORFeome grouped by phyla. Error bars represent 1 standard deviation. Download FIG S6, PDF file, 0.3 MB. Copyright © 2020 Villada et al. 2020 Villada et al. This content is distributed under the terms of the Creative Commons Attribution 4.0 International license.

Once we established that the bounded exponential model could be fitted to most *Bacteria* and *Archaea*, we evaluated the statistical significance of the modeling by estimating the *P* value for the rate parameter (a strong indicator of the ramp) in each successful fitted model (Fig. S7A). We found that most of the rate parameter estimates for *Bacteria* (99.5%) and *Archaea* (91%) were significant (*P < *0.001), while only eight were significant in the small subset of ORFeomes that were successfully modeled in *Fungi* (43 ORFeomes) (Fig. S7A). We further assessed whether the statistical significance of the rate parameter correlated with other molecular features (Fig. S7B). We found that the strongest correlation in *Bacteria* and *Archaea* was with the total length of the ORFeome and the number of CDSs per ORFeome (Pearson correlation coefficient, Fig. S7B). By linear regression modeling, we observed that ∼30% of the variation in the statistical significance of the rate parameter can be explained by the variation in the number of CDSs in the ORFeomes of *Bacteria* and *Archaea* (*R*^2^ = 0.35 with *P < *0.001 in *Bacteria* and *R*^2^ = 0.28 with *P < *0.001 in *Archaea*, Fig. S7C).

10.1128/mSystems.00613-20.7 FIG S7 Model fitting features of ORFeomes in the comprehensive data set. (A) Percentages of ORFeomes whose results were statistically significant (*P < *0.001) as modeled with the bounded exponential function. (B) Pearson correlation coefficient between the statistical significance of the rate parameter (−log_10_ of *P* value) estimated from the bounded exponential model and the different molecular features of ORFeomes analyzed in this study. (C) Linear regression model with the number of CDSs per genome as the independent variable and the statistical significance of the rate parameter (−log_10_ of *P* value) as the dependent variable. The gray-shaded region represents the 95% confidence interval of the regression model. Download FIG S7, PDF file, 0.3 MB. Copyright © 2020 Villada et al. 2020 Villada et al. This content is distributed under the terms of the Creative Commons Attribution 4.0 International license.

### Characteristics of the ramp of the number of hydrogen bonds in *Bacteria*, *Archaea*, and *Fungi*.

Further characterization of the bounded exponential ramp model parameters (Fig. 7C) showed that significant differences (*α*  = 0.1% was adopted for the analysis due to the large sample size) were not observed in the estimated parameter of carrying capacity of hydrogen bonds between *Bacteria*, *Archaea*, and *Fungi* (Fig. 7D, adjusted *P > *0.001). On the other hand, the estimated parameters of initial number of hydrogen bonds (Fig. 7E) and rate (Fig. 7F) were significantly different between all groups (adjusted *P < *0.001). We observed that the initial number of hydrogen bonds was lowest in *Bacteria* (Fig. 7E), which is consistent with the rate of increase in the number of hydrogen bonds per codon being the highest in *Bacteria* (Fig. 7F) to reach carrying capacities that were not significantly different between all groups after the ramp (Fig. 7D). Hence, by linear regression modeling between the estimated parameters for initial content and carrying capacity, one can approximate the rapidity of the change in the average number of hydrogen bonds per codon given that the carrying capacity becomes steady at about the 20th codon position (Fig. 7G).

We further assessed the phylogenetic relatedness of the ramp rate (the indicator for the existence of the ramp of hydrogen bonding) for the ORFeomes in the representative data set. A whole-genome phylogenetic tree was constructed, and the ramp rate was mapped to each branch (Fig. 7H). We observed that most of the phyla had similar median ramp rates (Fig. 7I), with *Actinobacteria*, *Proteobacteria*, *Verrumicrobia*, and *Bacteroidetes* showing the highest ramp rates among the bacterial phyla (Fig. 7I) and the phylum *Thaumarchaeota* having the highest ramp rates among the archaeal phyla (with a median value greater than that seen with some of the bacterial phyla) (Fig. 7I). Interestingly, the fungal phylum *Microsporidia* showed positive ramp rates and the median value was greater than that seen with some of the bacterial and archaeal phyla (Fig. 7I).

### A Web-based application to analyze position-dependent hydrogen bonding.

In order to facilitate the analysis of position-dependent hydrogen bonding of novel and custom ORFeomes, a Web-based graphical user interface (GUI) application was developed using the R package shiny (37). The application incorporates all the methods developed and implemented in this work. In a simple GUI (Fig. S8), the application allows interactive investigation of novel and customized ORFeomes, download of raw analysis and modeling data, and generation of high-quality figures. The application also reports summary statistics associated with modeling of hydrogen bonding per codon position by the bounded exponential model. For cases that cannot be successfully modeled, the application provides outputs that graphically represent the observed number of hydrogen bonds per codon position and a summary report of the analysis. The application is publicly available at https://juanvillada.shinyapps.io/hbonds/.

10.1128/mSystems.00613-20.8 FIG S8 Screenshot illustration of the Web-based graphical user interface application developed to analyze novel and customized ORFeomes. Examples of results of analysis of ORFeomes belonging to (A) *Bacteria* and (B) *Archaea* are shown. Download FIG S8, PDF file, 0.5 MB. Copyright © 2020 Villada et al. 2020 Villada et al. This content is distributed under the terms of the Creative Commons Attribution 4.0 International license.

## DISCUSSION

By first analyzing the ORFeome of E. coli as a model and subsequently over 14,000 bacterial, archaeal, and fungal ORFeomes, we found evidence for an exponential ramp of hydrogen bonding at the 5ʹ end of CDSs in *Bacteria* and *Archaea* that is created by a position-dependent codon usage bias. With the methods used in this investigation, a similar ramp in fungal ORFeomes was not identified. From a resource allocation perspective, a ramp of hydrogen bonding found in *Bacteria* and *Archaea* may provide an energy-efficient mechanism in which the energy required to melt hydrogen bonds (38–40) and unwind dsDNA is gradually increased. It has been reported previously that AU-rich codons are selected for at the beginning of CDSs in E. coli and other organisms (35) and that genomic GC content shows positional dependency in diverse organisms (41), which would in turn reduce the local hydrogen bonding at the 5ʹ end of CDSs. In contrast to previous studies where analyses were limited to characterizing only the first 15 to 20 codon positions (35) or a smaller set of ORFeomes (41), we analyzed a longer region of the 5ʹ end of CDSs (100 or 250 codon positions) and a data set with thousands of ORFeomes that included all three domains of life. Hence, we managed to identify parameters that mathematically describe the formation of the hydrogen bonding ramp and the extent of its conservation in microbial ORFeomes.

We have provided evidence indicating that the CDSs occupying the first position of operons in E. coli have lower levels of hydrogen bonding than internal CDSs and that this preference is the most obvious in the first ∼20 codons of the first CDS in an operon, suggesting that transcriptional efficiency might be favored at the beginning of the transcription unit (Fig. 2). By coupling hydrogen bonding and transcriptomics data of E. coli, we further showed that highly transcribed CDSs demand a lower maximum capacity of hydrogen bonds per codon, suggesting that the energetic requirement to unwind the dsDNA in highly transcribed CDSs has evolved to be minimized (Fig. 3). By contrasting position-dependent hydrogen bonding with codon usage metrics, we also showed selection acts to maintain tightly associated ramps in mRNA secondary structure and hydrogen boding in E. coli (Fig. 4) as well as generally in *Bacteria* and *Archaea* but not *Fungi* (Fig. 6). A parsimonious explanation for the existence of a ramp of hydrogen bonding in *Bacteria* and *Archaea*, but not *Fungi*, is that it is a molecular and evolutionary mechanism that optimizes the coupling of transcription and translation. Transcription and translation in *Bacteria* and *Archaea* are coupled in space and time (42), so the two processes influence each other. One such example can be found in the tight coordination maintained between transcription and translation in order to avoid premature termination of transcription (36). Therefore, it is reasonable to hypothesize that evolutionary traits may have been developed in order to optimally couple the transcription of protein-coding genes and the translation initiation of mRNA in *Bacteria* and *Archaea*. The ramp of hydrogen bonding might be one such trait that optimizes the efficiency of the coupling between transcription and translation (i.e., cotranscriptional translation efficiency) in *Bacteria* and *Archaea*. With a high level of cotranscriptional translation efficiency, dsDNA unwinding energy (i.e., hydrogen bonding) should be lower at the 5ʹ end of CDSs than at regions downstream of the start codon. Subsequently, efficient initial elongation of transcription occurs, and the nascent mRNA molecules effectively couple to the translation machinery such that translation elongation begins effectively. In turn, translation also follows a ramp of efficiency in which ribosomes are effectively recruited due to the relatively lower mRNA secondary structure, and initial elongation begins relatively slowly according to the enrichment of nonoptimal and rare codons at the 5ʹ end of CDSs (11, 43, 44).

In the proposed mechanism of cotranscriptional translation efficiency, although both transcription and translation seem to be mediated by an initial ramp, the ramps exhibit opposite efficiency. While a ramp of translation efficiency has been shown to start with a higher occurrence of nonoptimally translated codons at the 5ʹ end as a mechanism to possibly reduce traffic jams of ribosome downstream in translation elongation (5, 11, 45, 46), the ramp of hydrogen bonding found here at the same region starts with codons that reduce the energy required for unwinding dsDNA. Thus, the ramps of transcription and translation efficiency appear opposite but complementary in *Bacteria* and *Archaea*. This complementarity of speed can further reduce conflicts between the transcription and translation machineries (47).

From an evolutionary perspective, it will be interesting to further explore whether transcription or translation exerts a stronger selective pressure on local codon usage bias at the 5ʹ end of ORFeomes as the genomic evidence presented here do not allow distinguishing which mechanism drives selection. Nevertheless, the results presented here support the notion that the energy requirements for unwinding dsDNA of a CDS could be modulated by controlling the usage of synonymous codons to tune the number of hydrogen bonds. Although we found that the mean rate of increase of the number of hydrogen bonds per codon of *Bacteria* and *Archaea* is clearly higher than that of eukaryotes, some eukaryotes still showed a nonnegligible rate. We hypothesize that this may represent a signal of a remnant ramp that was lost in eukaryotes with the evolutionary emergence of packaged genomic DNA in the nucleus and further decoupling of transcription and translation. There is evidence in the literature showing that some nuclear sites can still support coupled transcription and translation in eukaryotes (48).

Most lines of evidence in this study resulted from focusing on the model organism E. coli. In the future, computational and experimental work should further investigate position-dependent hydrogen bonding in diverse genera in the tree of life. Future investigations should also consider integrating transcript and protein abundance data to investigate the role of position-dependent hydrogen bonding in the overall mechanism of protein biosynthesis.

Overall, we report the existence of a ramp of the number of hydrogen bonds that follows a bounded exponential function at the 5ʹ end of CDSs in *Bacteria* and *Archaea*. Optimization of cotranscriptional translation efficiency by reducing local hydrogen bonding can be another selective force driving the occurrence of AU-rich codons at the 5ʹ end of CDSs (35). The present work does not debunk any of the established translation-related and mRNA secondary structure-based theories of position-dependent codon usage bias (11, 12, 35). Instead, the ramp of hydrogen bonding encoded by a genomic signal adds another layer to the complexity of codon biology. The proposed mechanism for cotranscriptional translation efficiency might be another factor in the multiobjective optimization of gene expression, but more evidence is required. The genomic evidence compiled here suggests that effective coupling of transcription and translation at the 5ʹ end of CDSs of *Bacteria* and *Archaea* might be achieved by natural evolution via increasing the rate of occurrence of synonymous codons that also reduce hydrogen bonding, complementing the subtle effects of codons on the molecular biology of cells (2, 6, 13, 33, 45).

## MATERIALS AND METHODS

### ORFeomes, quality control of CDSs, and genomic analyses.

The ORFeome of Escherichia coli K-12 substrain MG1655 (4,141 CDSs) was used as the main showcase example. Other model ORFeomes investigated were those of the well-studied *Bacteria* “*Candidatus* Methylacidiphilum kamchatkense” Kam1 (2,196 CDSs), the *Archaea* Methanosarcina acetivorans (4,540 CDSs) and Haloferax volcanii (4,027 CDSs), the yeast Saccharomyces cerevisiae (6,705 CDSs) and Schizosaccharomyces pombe (5,147 CDSs), and the filamentous *Fungi* Neurospora crassa (11,653 CDSs) and Aspergillus nidulans (10,535 CDSs). The ORFeomes were retrieved from the *EnsemblBacteria* (bacteria.ensembl.org) and *EnsemblFungi* (fungi.ensembl.org) databases.

A comprehensive data set of ORFeomes (*n_total_* = 14,511), including 13,921 *Bacteria*, 297 *Archaea*, and 293 *Fungi*, were retrieved from the NCBI/RefSeq (49). The commands used to compile the ORFeomes were “*Latest RefSeq*” and “*Exclude anomalous*.” A smaller data set of representative ORFeomes (*n*_total_ = 1,766) was compiled that included all the *Bacteria* (*n *= 1,176) from a previously curated list that has even representation across phyla (18) and all the *Archaea* (*n *= 297) and *Fungi* (*n* = 293) ORFeomes in the comprehensive data set.

For all ORFeomes analyzed in this work, CDSs with lengths not divisible by 3 and shorter than the number of codons analyzed (100 or 250) were removed from the data set. The start codon was removed from the data set before conducting any downstream analyses. The length, GC content of each CDS, and GC content of each nucleotide position within a codon (GC_1_, GC_2_, and GC_3_) were calculated with SeqinR (50). Taxonomic affiliation of all downloaded ORFeomes was mapped using the XML file with the accession numbers of the ORFeomes and the table of lineages of all genomes deposited in NCBI. The table of lineages was generated using NCBItax2lin (https://github.com/zyxue/ncbitax2lin) with the NCBI taxonomy database (accessed February 2019). Information regarding the complete and representative ORFeome data sets can be found in Table S1 and Table S2 in the supplemental material, respectively.

10.1128/mSystems.00613-20.9 TABLE S1 Information corresponding to the comprehensive data set of ORFeomes analyzed in this study. Download Table S1, TXT file, 9.0 MB. Copyright © 2020 Villada et al. 2020 Villada et al. This content is distributed under the terms of the Creative Commons Attribution 4.0 International license.

10.1128/mSystems.00613-20.10 TABLE S2 Information corresponding to the representative data set of ORFeomes analyzed in this study. Download Table S2, CSV file, 0.05 MB. Copyright © 2020 Villada et al. 2020 Villada et al. This content is distributed under the terms of the Creative Commons Attribution 4.0 International license.

### Position-dependent number of hydrogen bonds.

DNA sequences were analyzed using the R packages Biostrings (51) and SeqinR (50). Nucleotides in each coding sequence were arranged in a matrix with dimensions equal to the number of CDSs as the number of rows and with the number of codons analyzed as the number of columns. After quality control, all the CDSs in an ORFeome were left aligned from the 5ʹ end. The number of hydrogen bonds was computed and stored in a matrix according to the nucleotide base composition of CDSs (adenine [A] = thymine [T] = 2; guanine [G] = cytosine [C] = 3). The number of hydrogen bonds at each codon position in an ORFeome was computed by calculating the mean and the 95% confidence interval of the mean with nonparametric bootstrapping (1,000 bootstraps) using the Hmisc (52) package in R. Matrix analysis and bootstrapping of thousands of ORFeomes were possible due to parallelization of the computational processes in multiple computer cores using the R packages foreach (53), doParallel (54), and doSNOW (55).

The relative number of hydrogen bonds was calculated as the observed content divided by the maximum number of hydrogen bond per amino acid. The scaled number of hydrogen bonds was calculated by centering and scaling the hydrogen bond contents of codons per amino acid using the scale function in R.

### Analysis of hydrogen bonding in operons.

Operons of E. coli K-12 substrain MG1655 were delineated using the Operon-mapper Web server (56). The DNA_topLevel genomic sequence FASTA and the GFF files from EnsemblBacteria (bacteria.ensembl.org) were used as input. The number of codons to analyze per CDS was set to 100, and the minimum number of CDSs per operon was set to 2. All CDSs in the ORFeome were categorized according to their position within the operons, and all CDSs located at the same operon position were aligned by the start codon. The number of hydrogen bonds in CDSs of operons was quantified (i) in separate regions of 20 codons up to the 100th codon position, (ii) from codon position 1 to position 100, and (iii) over the entire length of CDSs.

### Quantification of hydrogen bonding in highly and minimally expressed CDSs.

Transcriptomic data (48 independent sets) generated from 16 different RNA-Seq experiments using E. coli K-12 substrain MG1655 in triplicate (32) were downloaded from the Gene Expression Omnibus (accession no. GSE45443). The transcript abundance estimates (in reads per kilobase per million [RPKM]), calculated using Rockhopper software, were retrieved from the reference (32) and then mapped to the E. coli K-12 substrain MG1655 genomic sequences obtained from GenBank (accession no. U00096.3). CDSs in each of the 16 experiments were ranked according to their transcript abundances, and the CDSs that appeared in all 16 experiments at above or below the desired expression level threshold were grouped using the Reduce function in R for downstream quantification of hydrogen bonding. Six corresponding pairs corresponding to high expression threshold levels (i.e., top 5%, 10%, 15%, 20%, 25%, and 30%) and low expression threshold levels (i.e., bottom 13%, 18%, 23%, 26%, 30%, and 35%) were examined. The expression thresholds of the minimally expressed CDSs were set at levels that allowed similar numbers of CDSs to be compared against the corresponding highly expressed CDSs. The start codon was removed from all CDSs before quantification of the number of hydrogen bonds up to the 100th codon position. The mean number of hydrogen bonds per codon position of all CDS in each group was fitted with the locally estimated scatterplot smoothing (LOESS) nonparametric regression model.

### Position-dependent occurrence of frequent codons and optimal codons.

The position-dependent occurrences of frequent codons and of rare codons were computed with relative synonymous codon usage (RSCU) values (57), and the frequencies of optimal codons were computed with codon adaptation index (CAI) values (57). RSCU and CAI values were calculated as described previously (33) except that the geometric mean was not computed for each CDS. Instead, each codon was assigned a value according to the table of codon usage calculated with the function uco in SeqinR (50). By default, codons containing an undetermined nucleotide (N) were assigned the value “1” (no bias). RSCU and CAI values corresponding to every codon position of an ORFeome were calculated as the mean and the 95% confidence interval of the mean with nonparametric bootstrapping (1,000 bootstraps).

### Position-dependent translation efficiency.

Position-dependent translation efficiency was estimated with tRNA adaptation index (tAI) values (58). Position-dependent tAI values were calculated using the s vector as *s*_prokaryote_ = (0, 0, 0, 0, 0.41, 0.28, 0.9999, 0.68, 0.89) for *Bacteria* and *Archaea* and *s*_eukaryote_ = (0, 0, 0, 0, 0.41, 0.28, 0.9999, 0.68, 0.89) for *Fungi* as suggested previously (59). CodonR, the original algorithm used to compute tAI values (github.com/mariodosreis/tai), was customized in R to retrieve the value of every codon in a position-dependent manner. tRNA data sets for model organisms were obtained from the genomic tRNA database (GtRNAdb) (v2.0) (60) and the tRNA gene database curated by experts (tRNADB-CE) (v12.0) (61). The matrix of codon usage to compute tAI was obtained with CodonM (github.com/mariodosreis/tai/blob/master/misc/codonM). The parameter sking in the get_ws function was set to a value of 0 for eukaryotes and a value of 1 for *Bacteria* and *Archaea*. The tAI value in every codon position of an ORFeome was calculated as the mean and the 95% confidence interval of the mean with nonparametric bootstrapping (1,000 bootstraps).

### Position-dependent mRNA secondary structure.

The mRNA secondary structure was predicted by calculating the probability of a base being unpaired in the mRNA molecule using the program RNAplfold (v2.4.14) from the ViennaRNA package 2.0 (62) with the parameters *L *= 40, *W *= 40, and *u *= 40. Data representing secondary structure probabilities were parsed to R objects using a previously described method (63). The mean probability of each base being unpaired was calculated as the mean of all probabilities of a base being unpaired in any position, and the probability of a codon being unpaired was calculated as the mean of its number of bases. The probability of a codon forming a secondary structure in the mRNA molecule was calculated as the difference between 1 and its probability of being unpaired. The probability of a codon forming a secondary structure in every codon position of an ORFeome was calculated as the mean and the 95% confidence interval of the mean with nonparametric bootstrapping (1,000 bootstraps).

### Model fitting.

The uniform model [y(x)=A], linear model [y(x)=Bx+C], and bounded exponential model (equation 1) were used to model the mean number of hydrogen bonds per codon as a function of codon position (starting from the 2nd codon position).

(1) $$y\left(x\right)=\frac{\mathrm{AC}e^{\mathrm{Bx}}}{A+C\left[e^{\mathrm{Bx}}-1\right]}$$

In the models, *y* is the mean number of hydrogen bonds and *x* is the codon position; *A* is the carrying capacity of hydrogen bonds, defined as the maximum average number of hydrogen bonds that a particular codon position can contain in an ORFeome; *B* is the rate of hydrogen bonds per codon, defined as the change in the number of hydrogen bonds per codon; and *C* is the initial content, defined as the number of hydrogen bonds at the first codon after the start codon.

The models were fitted to hydrogen bonding data concerning the first 100 codon positions as the independent variable and the mean number of hydrogen bonds as the dependent variable. Self-Starting Nls Logistic Model was used to estimate the initial parameters, and weighted least-squares for a nonlinear model was used to estimate the final parameters (both were computed in R). As described previously (34), the Akaike information criterion (AIC) and Bayesian information criterion (BIC) were used to select the model that best fitted a data set. In cases in which the exponential model could not be successfully fitted but parameters were needed for downstream analyses, the initial content and carrying capacity parameters were calculated, respectively, as the minimal number of hydrogen bonds among all codon positions per ORFeome and the trimmed mean number of hydrogen bonds among all codon positions calculated after filtering out 20% of the codons (10 codons from each end).

### Phylogenomic analysis.

The translated CDSs of the representative data set of ORFeomes were used to construct a phylogenetic tree using the large-scale phylogenetic profiling of genomes method in PhyloPhlAn2 (bitbucket.org/nsegata/phylophlan/wiki/phylophlan2). The *supermatrix_aa* config file was used as input to build the tree with the parameters *diversity*=high and *database*=phylophlan. The ramp rates estimated from the exponential bounded model were mapped to each branch of the tree using ggtree (64) to integrate the phylogeny and hydrogen bonding parameters.

### Building the position-dependent null models of ORFeomes with shuffled codons.

The null model to test selection against uniform distribution of codons was built by shuffling synonymous codons within all CDSs in each ORFeome. A total of 200 simulated ORFeomes were built for each of the 1,496 ORFeomes (only *Bacteria* and *Archaea*) in the representative data set from which we obtained the metrics of expected and standard deviation of the number of hydrogen bonds per codon position as described in detail elsewhere (33). Having the observed and expected occurrence of the number of hydrogen bonds per codon, we then computed the *z*^2^ of the χ^2^ statistic as shown in equation 2:

(2) $${\text{χ}}^{2}=\overset{n}{\underset{i=1}{\sum }}\frac{{\left(O-E\right)}^{2}}{\sigma^{2}}=\overset{n}{\underset{i=1}{\sum }}z^{2}$$

where *O* is the observed count of the number of hydrogen bonds per codon position, *E* is the expected count of the number of hydrogen bonds per codon position computed from the 200 simulated ORFeomes, σ is the standard deviation of the number of hydrogen bonds per codon position computed from the 200 simulated ORFeomes, *n* is the number of codon positions, and *z* is the *z* score per codon position.

The hanging chi-gram (χ-gram) value per position is calculated as shown in equation 3. The parameters in equation 3 are as defined in equation 2.

(3) $${\text{χ}}_{\text{gram}}=\frac{O-E}{\sqrt{E}}$$

### Statistics, data analysis, and data visualization.

Data analysis was conducted in R (v3.6.0) using RStudio (v1.2.1335). The R package tidyverse (65) was used for data analytics, ggplot2 (66) for data visualization, and cowplot (67) for assembling multiple figure panels. Unless otherwise specified, differences between sample groups were tested using two-sided, nonpaired Wilcoxon rank sum test (Mann-Whitney test). The Kruskal-Wallis test was applied in the operon analysis to test the statistical significance of the differences in the number of hydrogen bonds between CDSs of each region. Correction of *P* values in multiple testing was done with the Benjamini and Yekutieli method (68). Pearson’s product-moment coefficient was used for linear correlation analyses, and Spearman’s ρ statistic was used to estimate a rank-based measure of association. Spearman’s ρ was also used in the correlation network analyses. A generalized additive model (GAM) was used to describe the position-dependent hydrogen bonding as a function of the probability of mRNA secondary structure formation. Scaled χ-gram values were calculated by centering and scaling each ORFeome. Normalized *z*^2^ values were computed using the *min-max* normalization function for each ORFeome (equation 4) as follows:

(4) $$y\left(x\right)=\frac{x-{\text{min}}_{x}}{{\text{max}}_{x}-{\text{min}}_{x}}$$

where *x* is the χ-gram value (equation 3), $\min_{x}$ is the minimum χ-gram value of an ORFeome, and $\max_{x}$ is the maximum χ-gram value of an ORFeome.

### Code and data availability.

The scripts required to reproduce all the results and figures can be obtained from https://github.com/PLeeLab/H_bonds_ramp. We developed a Web application (https://juanvillada.shinyapps.io/hbonds/) for users to analyze the position-dependent content of hydrogen bonding of ORFeomes.
