# Supplementary figures and images for: Interplay between Position-Dependent Codon Usage Bias and Hydrogen Bonding at the 5ʹ End of ORFeomes
Source: mSystems. 2020 Aug 11;5(4):e00613-20. doi: 10.1128/mSystems.00613-20 (PMC7426154; doi:10.1128/mSystems.00613-20)

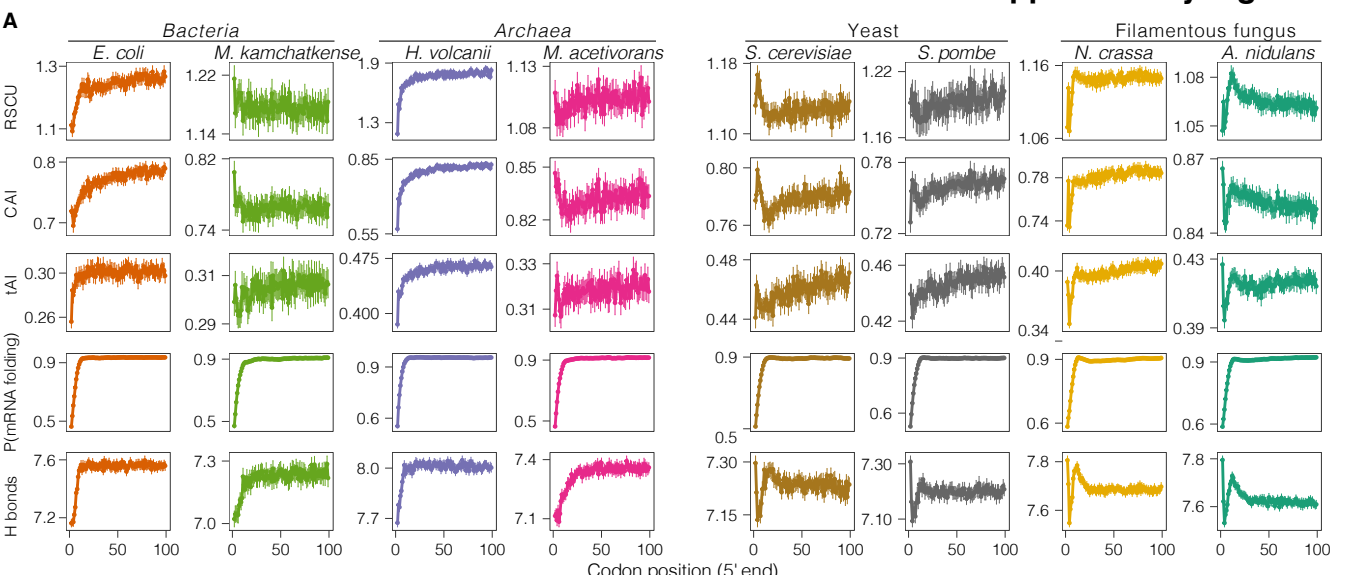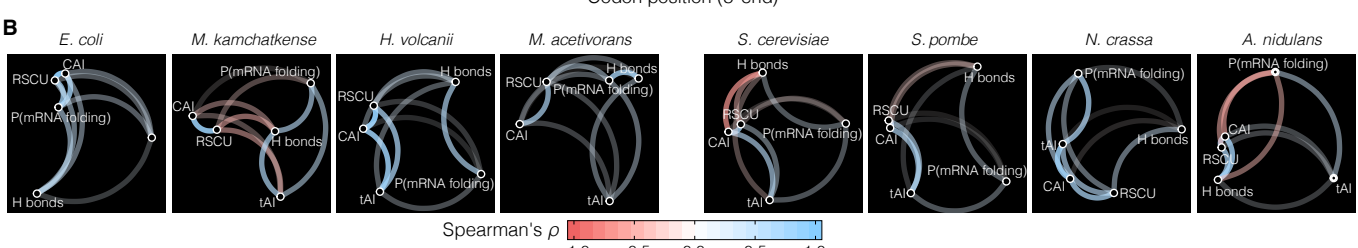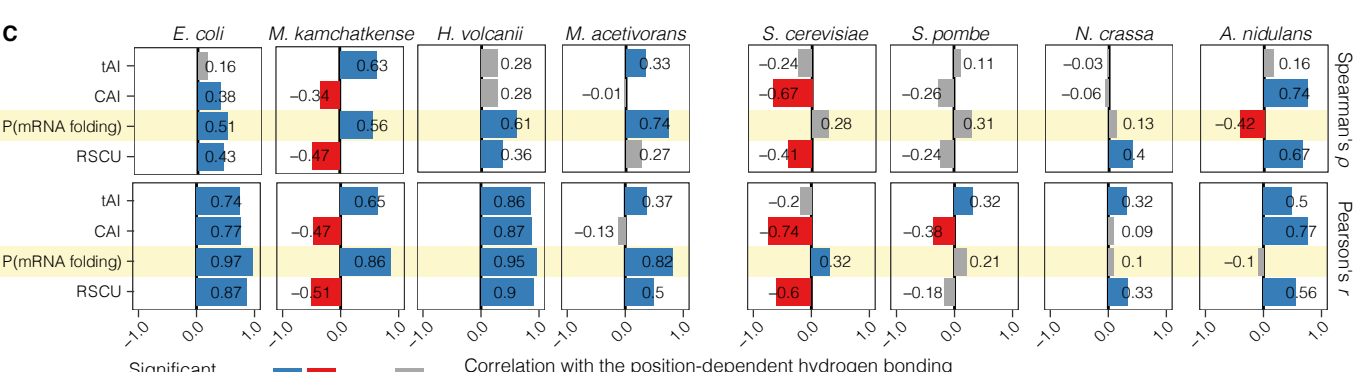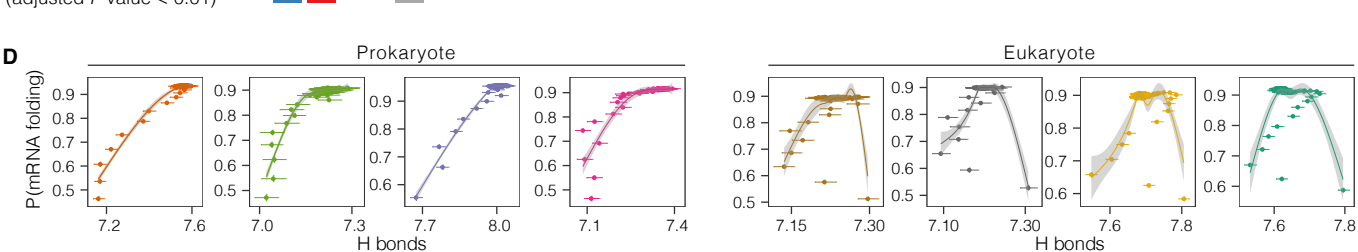

Supplement: FIG S1 [file mSystems.00613-20-sf001.pdf]

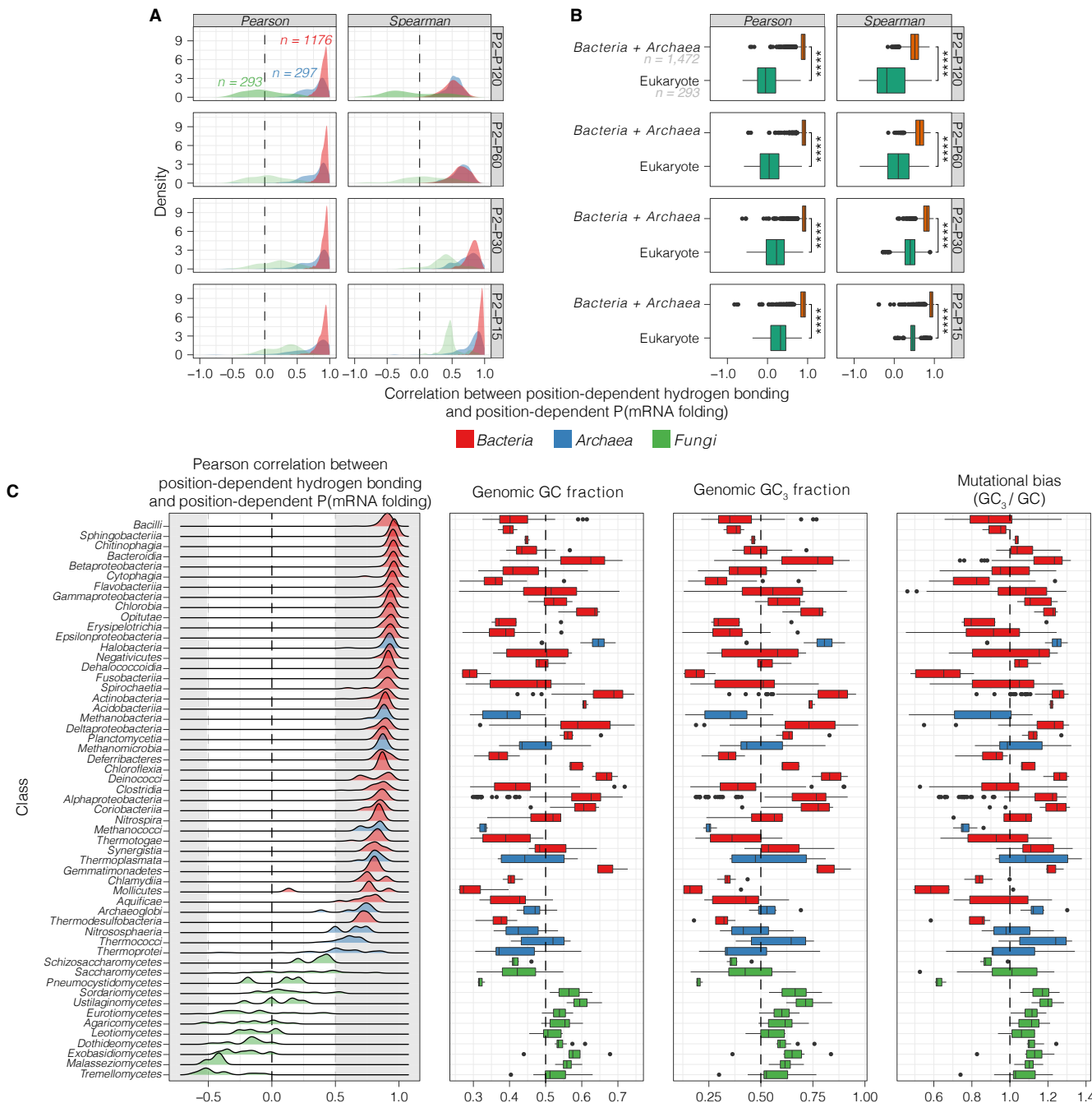

Supplement: FIG S2 [file mSystems.00613-20-sf002.pdf]

# Supplementary Figure S3

**A**

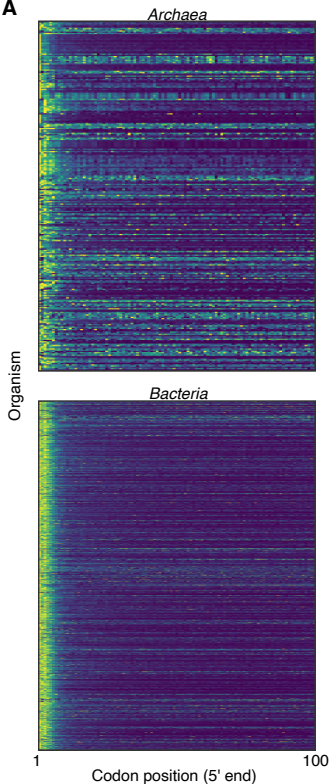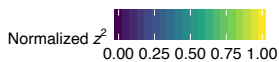

**B**

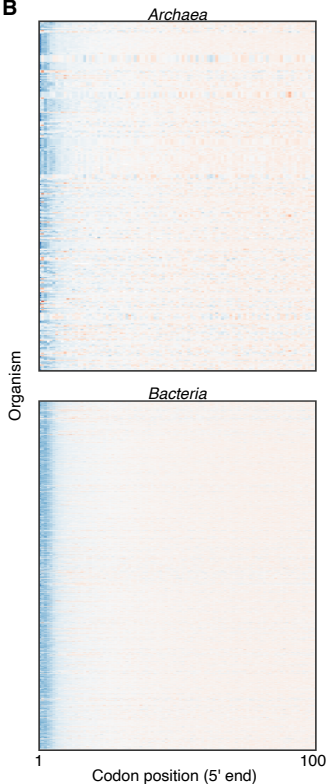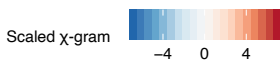

Supplement: FIG S3 [file mSystems.00613-20-sf003.pdf]

# Supplementary Figure S4

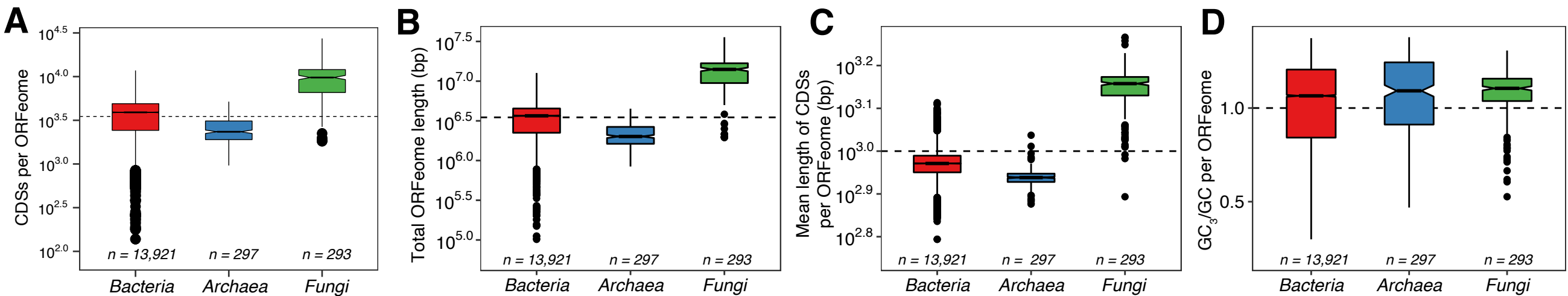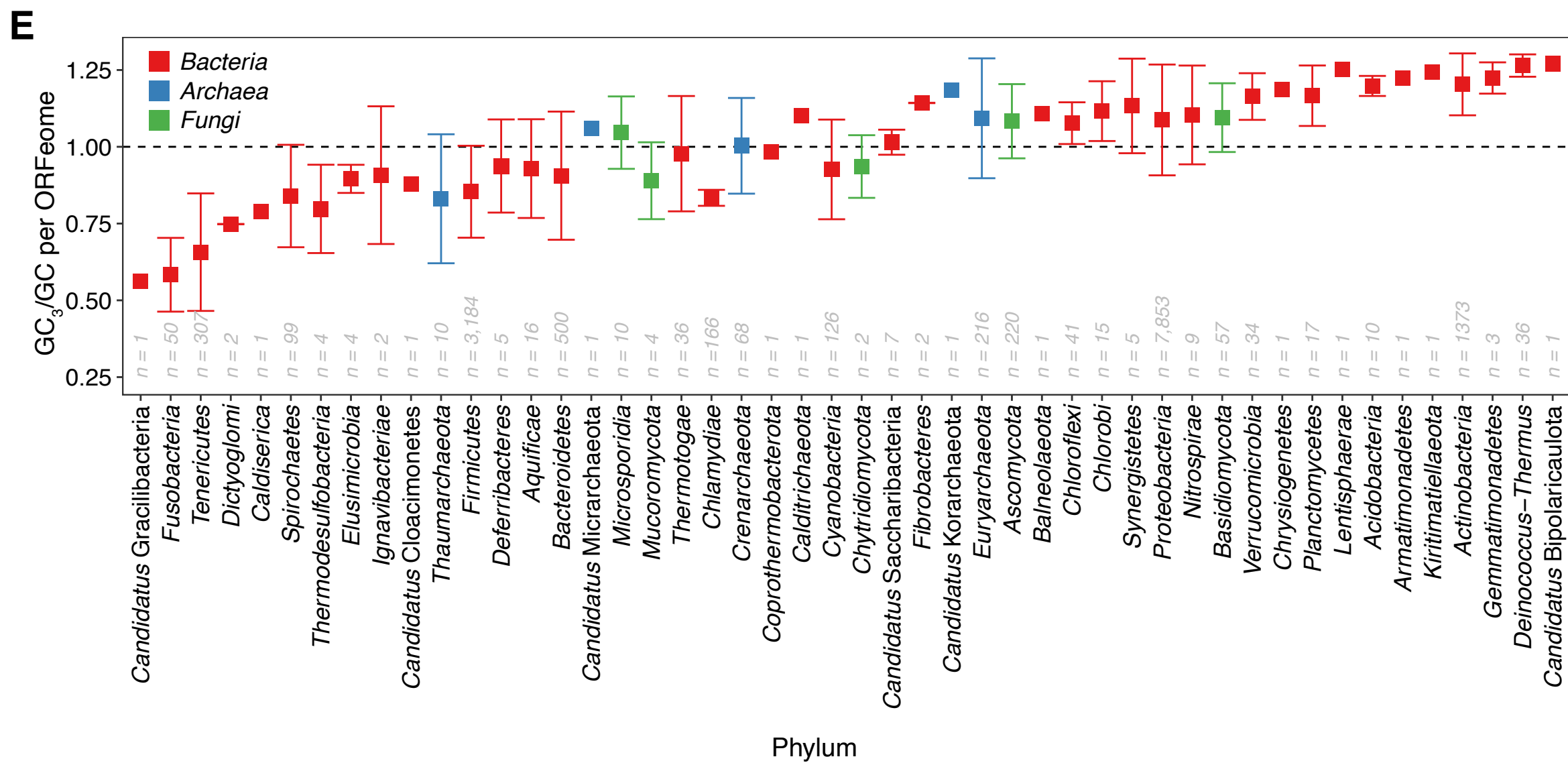

Supplement: FIG S4 [file mSystems.00613-20-sf004.pdf]

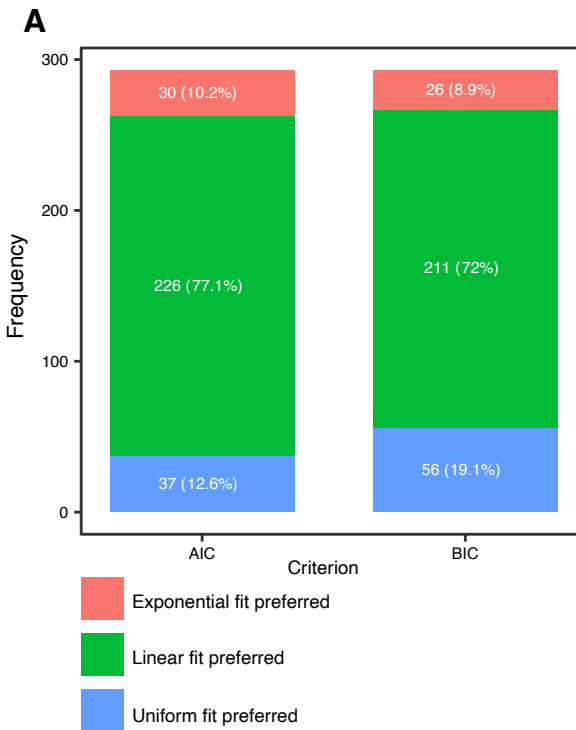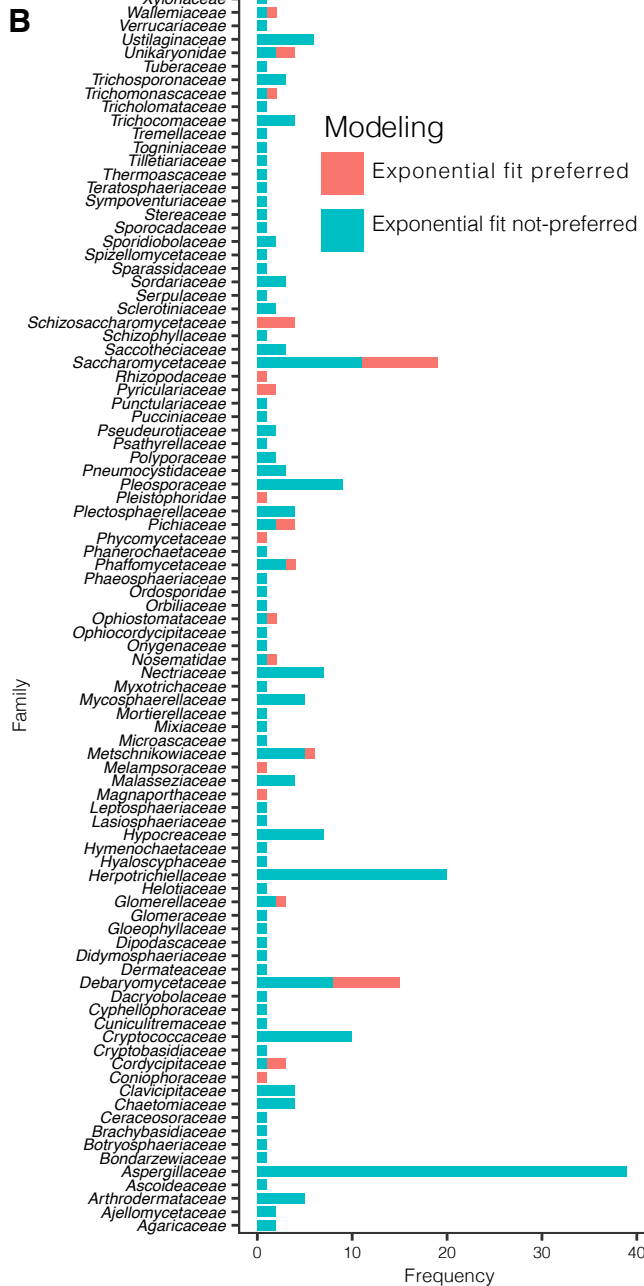

Supplement: FIG S5 [file mSystems.00613-20-sf005.pdf]

# Supplementary Figure S6

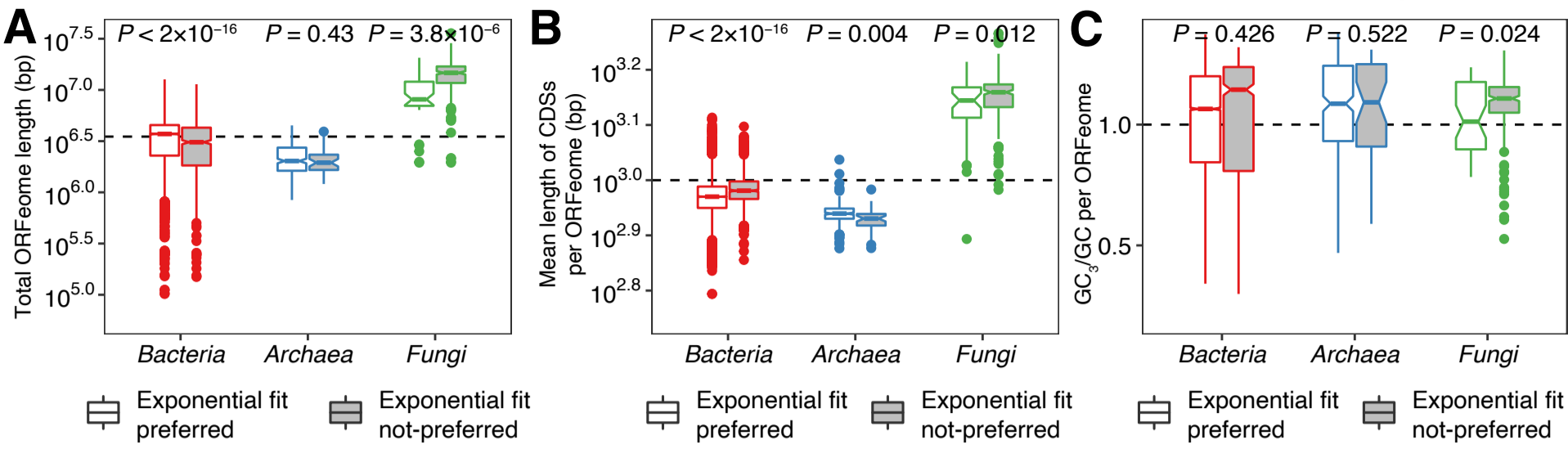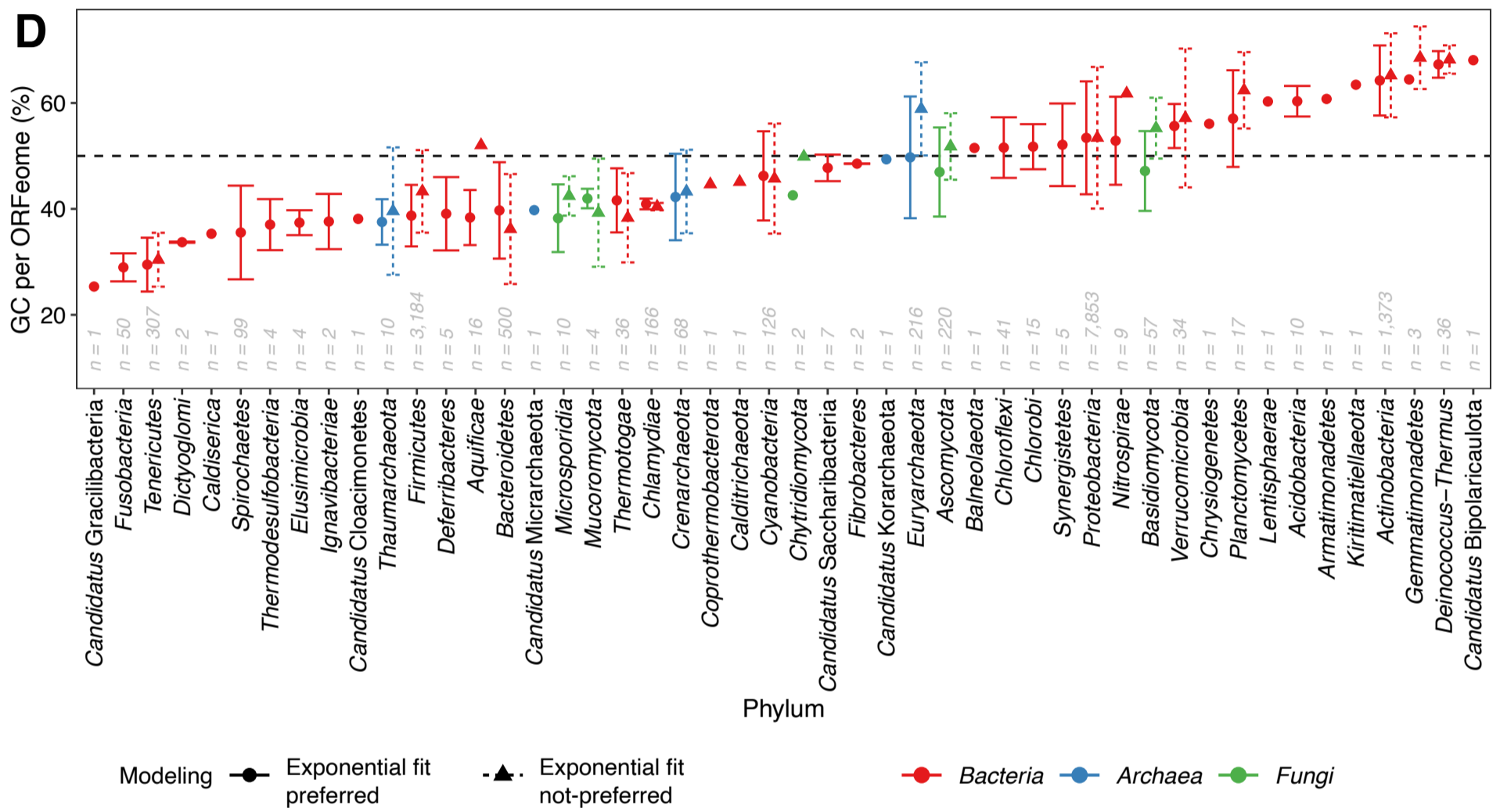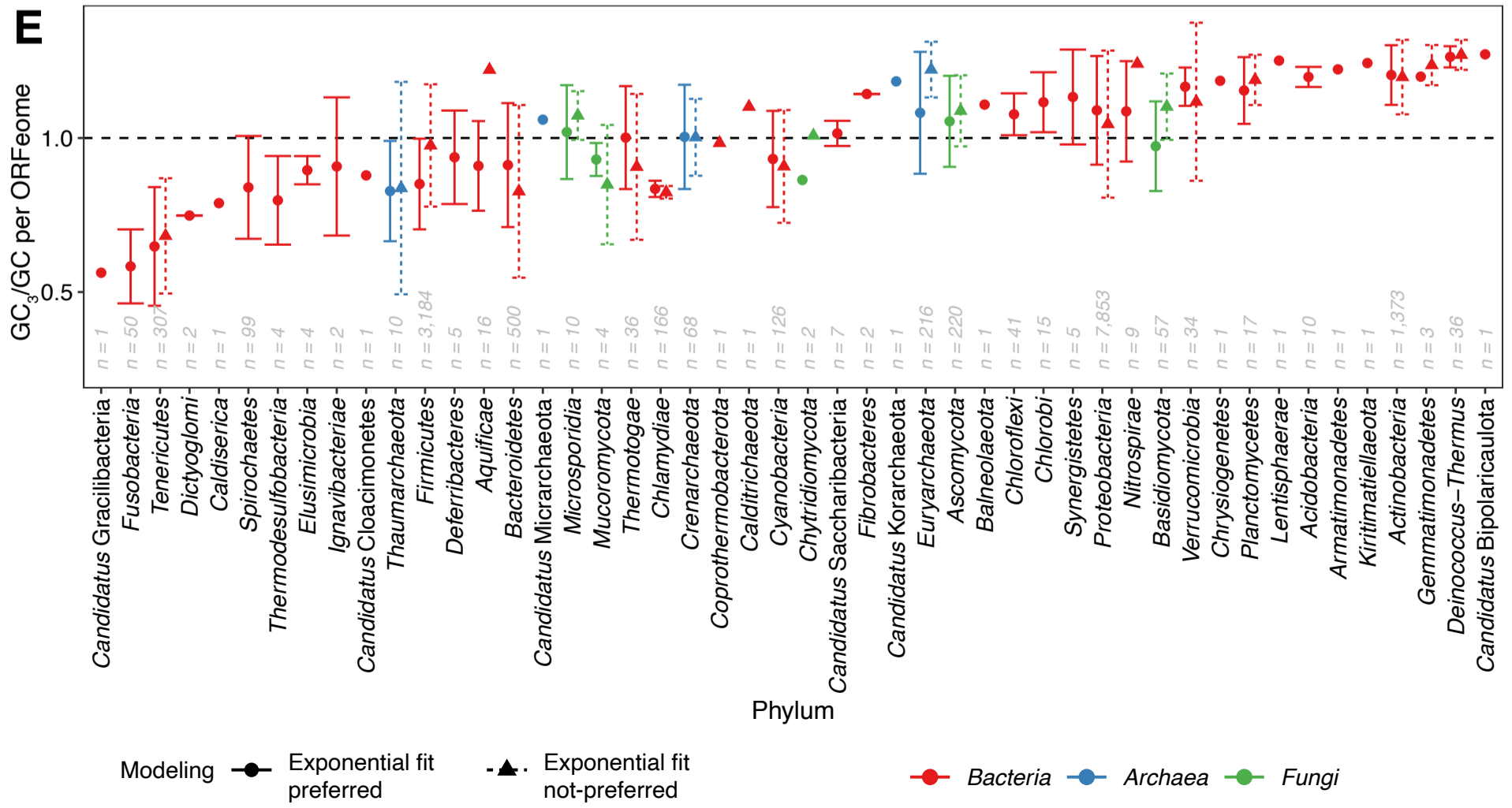

Supplement: FIG S6 [file mSystems.00613-20-sf006.pdf]
