# Supplementary material for: Interplay between Position-Dependent Codon Usage Bias and Hydrogen Bonding at the 5ʹ End of ORFeomes
Source: mSystems. 2020 Aug 11;5(4):e00613-20. doi: 10.1128/mSystems.00613-20 (PMC7426154; doi:10.1128/mSystems.00613-20)

Supplementary Figure S7

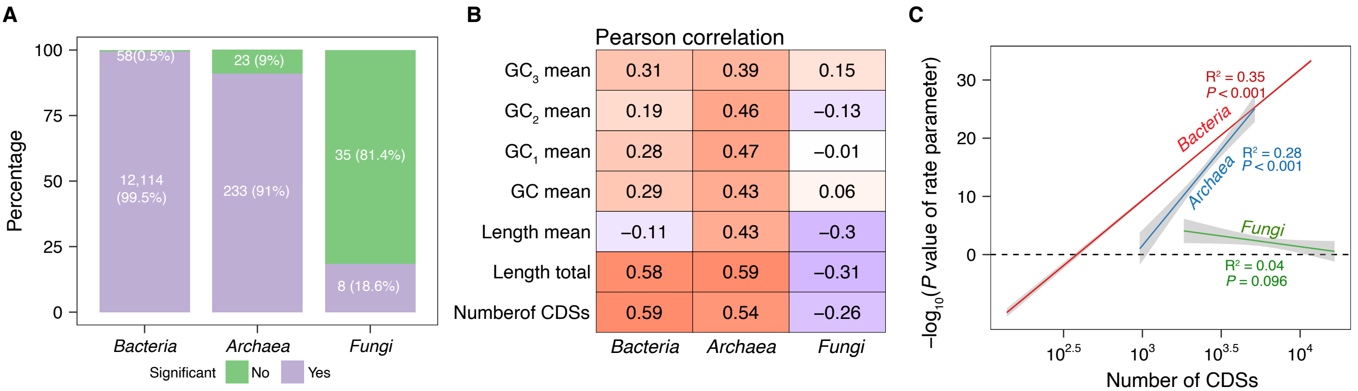

Supplement: FIG S7 [file mSystems.00613-20-sf007.pdf]
