# Supplementary material for: Interplay between Position-Dependent Codon Usage Bias and Hydrogen Bonding at the 5ʹ End of ORFeomes
Source: mSystems. 2020 Aug 11;5(4):e00613-20. doi: 10.1128/mSystems.00613-20 (PMC7426154; doi:10.1128/mSystems.00613-20)
Supplement: FIG S8 [file mSystems.00613-20-sf008.pdf]

Supplementary Figure S8

1. Upload your prokaryotic ORFeome

Use the `Browse` option to upload your file. The fasta file can be `gz` compressed or uncompressed. ORFeomes from sequenced prokaryotes can be downloaded from ENSEMBL selecting the **cDNA (FASTA)** column file

2. Start the analysis

Press the `Start Analysis` button and wait a few seconds

3. Results

Once the analysis is done, you should see a figure and table. You can download the main figure using the `Download plot` button. You can download the raw data to generate the figure using the `Download data` button.

4. Reference

Please cite this work as:

Villada JC, Duran MF, Lee PKH. Codon usage bias creates a ramp of hydrogen bonding at the 5'-end in prokaryotic ORFeomes (**2019**) *bioRxiv 811612*; doi: https://doi.org/10.1101/811612

Optional:

Download the Azotobacter vinelandii DJ ORFeome for test. The `gz` compressed `FASTA` file can be uploaded to the App.

Download test ORFeome

Upload ORFeome (6MB max, FASTA format, gzip supported)

Browse... No file selected

Start analysis

A

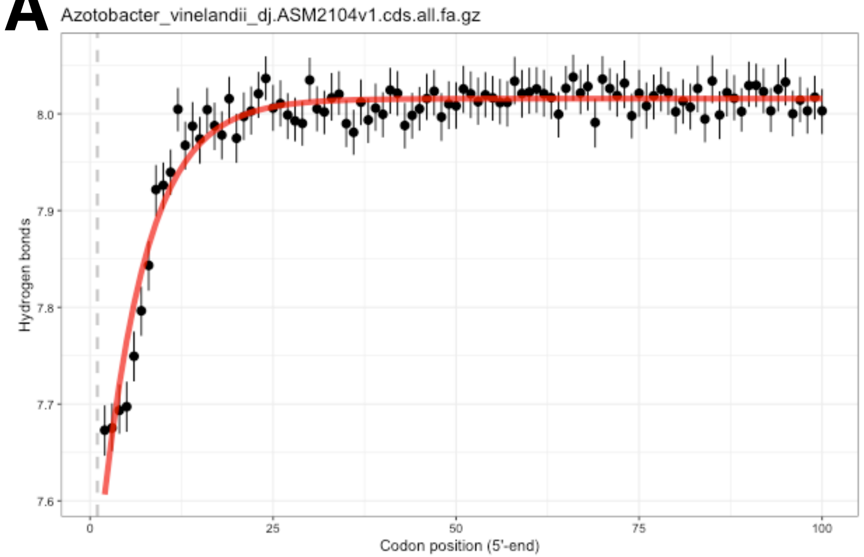

Download plot Download data

Feature	Value
ORFeome	Azotobacter_vinelandii_dj.ASM2104v1.cds.all.fa.gz
Modeling	Successful (Bounded Exponential Function)
Element	Hydrogen bonds
Carrying_capacity	8.015694
Initial_cost	7.451729
Rate	0.1706893
AIC	-489.7674
BIC	-479.387
Pval_Carrying_capacity	7.458646e-247
Pval_Rate	2.236754e-32
Pval_Initial_cost	1.935592e-142

B

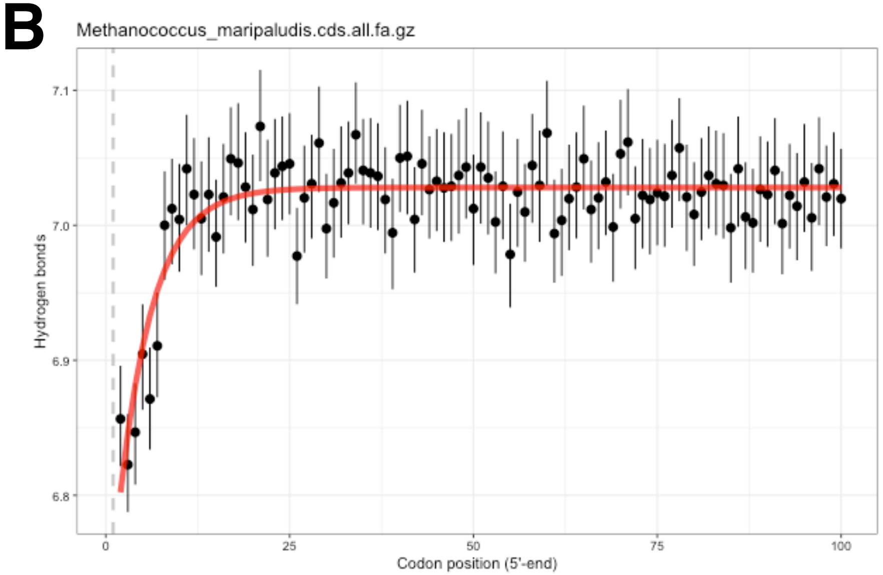

Download plot Download data

Feature	Value
ORFeome	Methanococcus_maripaludis.cds.all.fa.gz
Modeling	Successful (Bounded Exponential Function)
Element	Hydrogen bonds
Carrying_capacity	7.028009
Initial_cost	6.682433
Rate	0.2214133
AIC	-465.7225
BIC	-455.342
Pval_Carrying_capacity	8.992287e-238
Pval_Rate	3.396509e-12
Pval_Initial_cost	1.352656e-120
